# Analgesic Efficacy of Acetaminophen/Ibuprofen as an Adjuvant in Patient-Controlled Analgesia for Postoperative Pain After Oncoplastic Breast Surgery: A Randomized Controlled Trial

**DOI:** 10.3390/jcm14227901

**Published:** 2025-11-07

**Authors:** Jin Ha Park, Dong Won Lee, Eun Jung Kim, Jin Sun Cho

**Affiliations:** 1 Department of Anesthesiology and Pain Medicine, Yonsei University College of Medicine, Seoul 03722, Republic of Korea; realsummer@yuhs.ac (J.H.P.); natlis@yuhs.ac (E.J.K.); 2 Anesthesia and Pain Research Institute, Yonsei University College of Medicine, Seoul 03722, Republic of Korea; 3 Department of Plastic and Reconstructive Surgery, Institute for Human Tissue Restoration, Yonsei University College of Medicine, Seoul 03722, Republic of Korea; xyphoss@yuhs.ac

**Keywords:** acetaminophen, ibuprofen, multimodal analgesia, oncoplastic breast surgery, postoperative pain

## Abstract

**Background/Objectives**: Although patients with breast cancer often undergo multiple oncologic procedures after primary tumor resection, an optimal postoperative analgesic strategy remains undefined. We evaluated the efficacy of acetaminophen/ibuprofen as an adjunct to opioid-based patient-controlled analgesia (PCA) following oncoplastic breast surgery. **Methods**: In this double-blind randomized controlled trial, 79 patients were assigned to receive either acetaminophen/ibuprofen or saline. A 100 mL solution containing 1000 mg acetaminophen and 300 mg ibuprofen was administered at the end of surgery, and 200 mL was incorporated into a fentanyl-based PCA for infusion over 48 h. The control group received an equivalent volume of saline. The primary outcome was pain intensity at 1 h postoperatively, assessed using an 11-point numerical rating scale. Secondary outcomes included pain scores at 6, 24, and 48 h, cumulative fentanyl consumption via PCA, additional analgesic use, and adverse effects. **Results**: Pain scores at 1 h postoperatively were significantly lower in the intervention group than in the control group (median [IQR], 2 [2, 2] vs. 2 [2, 3], *p* = 0.040). Cumulative fentanyl volume administered via PCA was lower in the intervention group at 24 h (252.4 [186.7, 289.9] mcg vs. 299.7 [208.3, 366.6] mcg, *p* < 0.001) and 48 h (482.4 [283.2, 548.0] mcg vs. 537.0 [390.9, 586.0] mcg, *p* = 0.001). Fewer patients in the intervention group required rescue analgesics during the first 6 h (22 [56.4%] vs. 32 [80.0%], *p* = 0.024). Pain scores and rescue analgesic use thereafter did not differ between groups. **Conclusions**: Adjunctive acetaminophen/ibuprofen with opioid-based PCA reduced early postoperative pain, opioid consumption, and rescue analgesia without increasing adverse effects.

## 1. Introduction

Breast cancer is the most commonly diagnosed cancer among women worldwide, accounting for 23.8% of all cancers in females [1]. Even after tumor resection or mastectomy—the primary curative treatment—many patients undergo additional oncoplastic procedures and experience significant postoperative pain. Inadequate pain control increases the risk of complications, delays recovery, and contributes to chronic pain and a long-term decline in quality of life [2]. Therefore, it is crucial to establish an effective pain management strategy that minimizes adverse effects while supporting immune function in immunocompromised cancer patients.

Postoperative analgesic protocols after breast surgery vary and include systemic opioids, non-opioid analgesics, and regional techniques [3]. The Procedure-Specific Postoperative Pain Management (PROSPECT) guidelines for oncologic breast surgery recommend multimodal analgesia with a combination of paracetamol and nonsteroidal anti-inflammatory drugs (NSAIDs) as the first-line regimen, reserving opioids for rescue analgesia [3]. Similarly, the Enhanced Recovery After Surgery (ERAS) pathway emphasizes multimodal analgesia to improve care quality, facilitate return to preoperative conditions, and reduce opioid use [4]. Paracetamol and NSAIDs have been shown to reduce postoperative pain, opioid consumption, and opioid-related side effects [5,6]. Furthermore, perioperative NSAID use may enhance survival and reduce cancer recurrence [7,8]. Despite these advantages, opioids remain the mainstay of postoperative pain management after cancer surgery owing to their strong analgesic effects [9]. However, they have significant adverse effects, such as nausea, vomiting, hypotension, and an increased risk of immunosuppression [10]. Growing concerns regarding opioid epidemics and misuse have prompted reexamination of their role in postoperative pain management [11].

Although recommended by the PROSPECT guidelines, no prospective study has yet evaluated the efficacy and safety of the paracetamol/NSAID combination in patients undergoing oncoplastic breast surgery. Its routine use in this context remains unestablished. We aimed to assess the impact of adding a paracetamol/NSAID combination to standard opioid-based analgesia on pain intensity, opioid consumption, need for rescue analgesia, and safety compared to opioid-based analgesia alone.

## 2. Materials and Methods

### 2.1. Study Design and Participants

This study was approved by the Institutional Review Board and Ethics Committee of Severance Hospital, Yonsei University College of Medicine (#4-2022-1650) on 27 February 2023 and registered at clinicaltrial.gov (NCT06174363) on 18 December 2023. It was conducted in accordance with the Declaration of Helsinki, and the manuscript adheres to the Recommendations for the Conduct, Reporting, Editing, and Publication of Scholarly Work in Medical Journals.

Eligible patients were 20–70 years of age, classified as American Society of Anesthesiologists (ASA) physical status I–III, and scheduled for elective oncoplastic breast surgery following mastectomy between December 2023 and December 2024. Exclusion criteria were: known allergy to opioids, NSAIDs, or acetaminophen; severe cardiac dysfunction or hepatic/renal impairment (ASA class IV); history of gastrointestinal ulcers or bleeding; current aspirin use; asthma; inability to read or understand the informed consent form; and pregnancy or lactation. Written informed consent was obtained from all participants before enrollment.

### 2.2. Data Collection

Baseline characteristics, including demographics, comorbidities, and ASA physical status classification, were recorded. Preoperative laboratory assessments included serum creatinine and liver function tests. Intraoperative data included surgery type, durations of anesthesia and surgery, and total remifentanil use.

### 2.3. Interventions

After enrollment, patients were randomly assigned in a 1:1 ratio to receive either intravenous acetaminophen and ibuprofen (intervention group) or saline (control group) using a computer-generated random number table, without any stratification or blocking. Group allocation was concealed in sealed envelopes and neither the enrolling personnel nor those assigning participants to groups had access to the random allocation sequence. The intervention group received 100 mL of intravenous solution containing 1000 mg acetaminophen and 300 mg ibuprofen (Maxigesic^®^ IV, Kyungbo Pharm, Asan, Republic of Korea) over 15 min before the end of surgery. An additional 200 mL of the same solution was mixed into the fentanyl-based patient-controlled analgesia (PCA) for continuous infusion over 48 h. The control group received a matching volume of normal saline. The acetaminophen/ibuprofen combination and saline were prepared in identical syringes by a nurse who was not involved in this study. The solutions were indistinguishable in appearance to ensure blinding of care providers, investigators, patients, and outcome assessors. Group assignments were not revealed until patients were discharged.

### 2.4. Perioperative Management

In the operating room, all patients underwent routine monitoring, including electrocardiography, pulse oximetry, and noninvasive blood pressure measurement, in accordance with institutional protocols. No premedication was administered. General anesthesia was induced without neuraxial or regional blocks. Induction was achieved using intravenous propofol (1.5–2 mg/kg) and remifentanil (1 mcg/kg), followed by rocuronium (0.6 mg/kg) to facilitate tracheal intubation. Anesthesia was maintained with desflurane (4–7 vol%) and a continuous intravenous infusion of remifentanil (0.05–0.1 mcg/kg/min). The depth of anesthesia was adjusted to maintain a bispectral index (BIS) between 40 and 60, and mean arterial pressure was maintained within 20% of the pre-induction value. Intraoperative body temperature was maintained at 36.5 ± 0.5 °C. Approximately 15 min before the end of surgery, fentanyl (50 mcg) and ramosetron (0.3 mg) were administered for postoperative analgesia and nausea prophylaxis, respectively. Simultaneously, patients received 100 mL of the assigned study solution (either acetaminophen/ibuprofen or saline). Upon completion of surgery, all anesthetic agents were discontinued, and neuromuscular blockade was reversed using neostigmine (1 mg) and glycopyrrolate (0.2 mg). Extubation was performed once the patient regained consciousness and exhibited adequate spontaneous ventilation. No anti-inflammatory agents such as dexamethasone were administered perioperatively.

Patients underwent oncoplastic surgery classified as complexity 3 procedures [12], including delayed prosthetic reconstruction after mastectomy (placement of a tissue expander or permanent implant) and implant-related procedures (tissue expander removal and placement of a permanent implant, or implant exchange and breast remodeling in capsular contracture). Complex oncoplastic or reconstructive surgeries, such as flap surgery or reduction mammoplasty (Complexity 4 or higher), were excluded, as their higher complexity can significantly affect postoperative pain.

For postoperative analgesia, all patients received intravenous PCA for 48 h after surgery. At our institution, the standard analgesic regimen for oncoplastic breast surgery includes intravenous fentanyl (50 mcg) administered at the end of surgery, followed by fentanyl-based PCA. The PCA solution contained fentanyl (10 mcg/kg) and ramosetron (0.3 mg), diluted with saline to a total volume of 50 mL. An additional 200 mL of the allocated study solution was added, resulting in a total PCA volume of 250 mL. The PCA was set to deliver a basal rate of 5 mL/h, a bolus dose of 0.5 mL, and a lockout interval of 15 min. During the postoperative period, both tramadol and acetaminophen were available to all patients under the same standardized protocol. Patients with a pain score ≥ 4 on the 11-point numerical rating scale (NRS) received intravenous tramadol (50 mg) as rescue analgesia, while those with a score < 4 received intravenous acetaminophen (1000 mg) upon request.

### 2.5. Outcome Measures

The primary outcome was the intensity of acute postoperative pain, assessed using an 11-point NRS at rest and during movement, 1 h after surgery. Secondary outcomes included NRS pain scores at 6, 24, and 48 h postoperatively; cumulative fentanyl consumption via intravenous PCA during the first 24 and 48 h; the need for additional analgesics beyond PCA at 6, 24, and 48 h postoperatively; the incidence of analgesic-related adverse effects—including nausea, vomiting, headache, dizziness, and hypotension—and the length of hospital stay. All outcomes were assessed by clinicians who were blinded to group allocation.

### 2.6. Statistics

As no previous studies have investigated the effect of an acetaminophen/ibuprofen combination as an adjunct to opioid-based analgesia in patients undergoing oncoplastic breast surgery, the sample size was calculated based on a pilot study conducted at our institution involving 10 such patients. In patients who received fentanyl-based analgesia, the mean NRS pain score during movement 1 h postoperatively—the most painful time point—was 5.2, with a standard deviation of 1.5. Assuming a minimum clinically important difference of at least 1 point [13,14], with a significance level (α) of 0.05 and a power of 90%, the required sample size was estimated to be 37 patients per group. To account for an anticipated 10% dropout rate, we planned to enroll 42 patients in each group.

The normality of continuous variables was assessed using the Kolmogorov–Smirnov test. Based on data distribution, continuous variables were compared using either the two-sample *t* test (for normally distributed data) or the Mann–Whitney *U* test (for non-normally distributed data), and are reported as mean ± standard deviation or median (interquartile range), as appropriate. Categorical variables were compared using the chi-squared test or Fisher’s exact test and are presented as absolute numbers (percentages). All statistical analyses were performed using SPSS version 25.0 (IBM Corp., Armonk, NY, USA). A *p*-value < 0.05 was considered statistically significant.

## 3. Results

**Figure 1:**
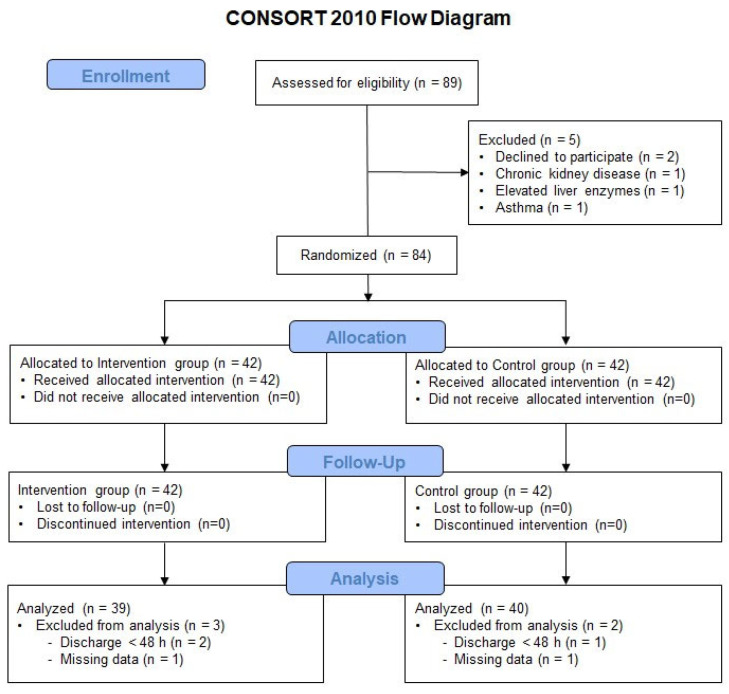
Flow diagram.

Of the 89 patients assessed for eligibility, five were excluded for the following reasons: declined to participate (n = 2), chronic kidney disease (n = 1), elevated liver enzymes (n = 1), and asthma (n = 1). A total of 84 patients were enrolled and randomly assigned to the intervention or control group. Among them, five patients were excluded from the final analysis: three were discharged within 48 h after surgery, and two had missing primary outcome data owing to incomplete pain assessments. Ultimately, 79 patients were included in the final analysis (Figure 1).

### 3.1. Demographics and Clinical Data

Baseline demographic and clinical data are presented in Table 1. There were no significant differences between the groups in terms of patient characteristics, type or distribution of surgery, duration of surgery or anesthesia, or intraoperative remifentanil consumption. Implant insertion was the most common procedure, followed by capsulectomy, tissue expander placement, and breast augmentation or reduction.

### 3.2. Postoperative Pain Intensity and Additional Analgesic Requirement

Postoperative pain intensity was assessed using the 11-point NRS at rest and during movement at 1, 6, 24, and 48 h after surgery. At 1 h postoperatively, the pain score during movement was significantly lower in the intervention group compared with the control group (median [IQR], 2 [2, 2] vs. 2 [2, 3]), with an absolute median difference of 0 (95% CI, −1.0 to 0; *p* = 0.040). Pain scores at all other time points did not differ significantly between groups (Table 2).

Cumulative fentanyl volume administered via PCA was significantly lower in the intervention group during the first 24 h (252.4 [186.7, 289.9] mcg vs. 299.7 [208.3, 366.6] mcg), with median difference of −49.7 (95% CI −92.3 to −4.1, *p* < 0.001) and over 48 h (482.4 [283.2, 548.0] mcg vs. 537.0 [390.9, 586.0] mcg), with median difference of −52.7 (95% CI, −149.0 to −21.0, *p* = 0.001). The need for additional analgesics beyond PCA was assessed at the following postoperative intervals: 0–6 h, 6–24 h, and 24–48 h. During the first six postoperative hours, a significantly higher proportion of patients in the control group required rescue analgesics than those in the intervention group (32 [80.0%] vs. 22 [56.4%], *p* = 0.024). Although the number of patients receiving tramadol (50 mg for NRS ≥ 4) or acetaminophen (1 g for NRS < 4 and upon patient request) individually and the corresponding doses did not differ between groups, the overall need for additional analgesics beyond PCA was significantly greater in the control group. No significant differences were observed between groups during the later time intervals (Table 3). In addition, a sensitivity analysis excluding patients who received tramadol rescue showed consistent results, supporting the robustness of the opioid-sparing effect. Cumulative fentanyl consumption was also lower in the intervention group during the first 24 h (257.3 [196.2, 310.1] mcg vs. 319.6 [217.2, 383.8] mcg, *p* = 0.002) and during the first 48 h (500.0 [278.4, 605.0] mcg vs. 544.0 [390.9, 602.0] mcg, *p* = 0.003).

### 3.3. Analgesic-Related Side Effects and Hospital Stay

There were no significant differences between groups in the incidence of analgesic-related side effects, including nausea/vomiting, dizziness, headache, hypotension, and pruritus. The length of hospital stay was also similar between the two groups (Table 4).

## 4. Discussion

In this randomized controlled trial, the addition of intravenous acetaminophen and ibuprofen to fentanyl-based PCA significantly reduced immediate postoperative pain intensity, opioid consumption, and the need for additional analgesics following oncoplastic breast surgery, without increasing adverse effects. These findings support the use of multimodal analgesia incorporating paracetamol and NSAIDs in accordance with PROSPECT guidelines.

Although surgical resection is essential for cancer treatment, it may inadvertently promote the dissemination of cancer cells due to physical manipulation and disruption of tissue integrity [15]. Surgery induces a pro-inflammatory response, followed by a compensatory anti-inflammatory phase, which can result in immunosuppression. This immunosuppressive state may be further exacerbated by sympathetic nervous system activation and the use of anesthetics and opioids [16]. Inadequate pain control can also worsen immune dysfunction, as pain itself is a potent immunosuppressive factor [16]. These perioperative immunological changes may create a window of vulnerability during which residual cancer cells can evade immune surveillance and proliferate, potentially leading to recurrence or metastasis [17]. Therefore, it is essential to adopt analgesic strategies that not only ensure effective pain control but also minimize immunosuppression.

Postoperative pain management should adopt a multimodal approach, using multiple pharmacological classes of analgesics to target different pain pathways. This strategy enhances analgesic efficacy and reduces the side effects associated with individual drugs [18]. The PROSPECT guidelines for oncologic breast surgery recommend administering a combination of paracetamol and either conventional NSAIDs or cyclooxygenase (COX)-2 selective inhibitors both preoperatively and intraoperatively, with continued use throughout the postoperative period as first-line analgesia. Opioids should be reserved for rescue use postoperatively, and regional analgesic techniques are recommended for major procedures [3]. The analgesic efficacy and opioid-sparing effects of paracetamol and NSAIDs are well-established across various perioperative settings, including breast cancer surgery [5,6,19]. Although opioids remain effective for managing acute pain after cancer surgery, they exert dose-dependent immunosuppressive effects by suppressing natural killer cell activity, cytokine production, and antibody formation [20,21]. These immune-modulating actions may contribute to enhanced breast cancer progression and metastasis [21,22]. Given these concerns, it is essential to explore analgesic strategies that minimize opioid use while ensuring adequate pain control in this vulnerable population, particularly in those undergoing multiple oncologic or oncoplastic surgeries throughout their lives.

NSAIDs and paracetamol enhance analgesic efficacy and reduce opioid requirements through distinct but complementary mechanisms. NSAIDs exert peripheral effects via their analgesic, antipyretic, and anti-inflammatory properties. They inhibit COX enzymes, thereby reducing prostaglandin synthesis and blocking inflammatory pathways [18]. Given the interplay between nociception and inflammation, NSAIDs provide effective analgesia by targeting both processes [23]. In addition, through COX-2 inhibition, NSAIDs may exhibit antitumor properties. While COX-2 is not expressed in normal breast tissue, it is overexpressed in up to 80% of breast tumors, leading to increased prostaglandin E2 synthesis—a potent mitogen that stimulates estrogen production and promotes tumor progression [24,25]. NSAIDs may counteract these effects by reducing prostaglandin levels, inhibiting aromatase activity, and lowering serum estrogen concentrations. Through these mechanisms, they may decrease the risk of hormone receptor-positive tumors and potentially contribute to anticancer effects by promoting apoptosis and limiting mutagenesis [26]. In breast cancer surgery, perioperative NSAID use has been associated with lower recurrence rates and improved disease-free and overall survival [27,28,29]. Paracetamol possesses analgesic and antipyretic properties similar to NSAIDs but lacks peripheral anti-inflammatory activity. Its central effects are thought to involve modulation of descending inhibitory pain pathways, and its peripheral effects involve the inhibition of prostaglandin synthesis within the CNS, likely via indirect COX inhibition. As paracetamol acts through mechanisms partly independent of COX, its combination with NSAIDs may provide additive analgesia by engaging distinct pathways [30]. This combination offers superior pain relief and greater opioid-sparing effects than either agent alone [31]. The fixed-dose combination used in this study has been safely administered and has demonstrated enhanced analgesic efficacy compared to either drug alone or placebo [32,33]. Additionally, when incorporated into opioid-based PCA, it significantly reduced opioid consumption [6].

Although systematic reviews support the enhanced efficacy of paracetamol and NSAID combination, findings have been inconsistent, and the reasons for this variability remain unclear [34]. The PROSPECT guidelines strongly recommend the use of paracetamol and NSAIDs for breast cancer surgery; however, few prospective studies have directly evaluated their combined efficacy. Most supporting evidence comes from comparisons of paracetamol with placebo [35,36], paracetamol/ibuprofen with paracetamol/codeine/caffeine [37], or NSAIDs added to paravertebral blocks [38], rather than studies directly comparing the paracetamol/NSAID combination with placebo or individual agents. The lack of high-quality comparative studies highlights the need for well-designed prospective trials to confirm the analgesic benefits of this regimen. Furthermore, despite the established advantages of multimodal analgesia, its implementation remains inconsistent across institutions, providers, and medication protocols [39]. To address this gap, we conducted a study to evaluate the efficacy and safety of adding paracetamol and NSAIDs to opioid-based postoperative analgesia. The addition of acetaminophen/ibuprofen to fentanyl-based PCA significantly reduced immediate postoperative pain during movement, decreased cumulative fentanyl consumption during 24 and 48 h, and reduced the need for additional analgesics within the first 6 h postoperatively. These findings support the routine use of a paracetamol/NASID combination as a part of multimodal analgesia, providing improved pain control and reduced opioid consumption without increasing adverse effects.

This study had some limitations. First, it was conducted at a single center, which may limit the generalizability of the findings. Second, both groups received fentanyl-based PCA with background infusion, which is standard practice at our institution to provide continuous low-dose opioid support for postoperative pain management. This may have attenuated the between-group differences in postoperative pain and opioid-related adverse events. Continuous opioid infusion may mask the opioid-sparing effects of adjunctive analgesics. A previous study comparing fentanyl-based PCA with and without background infusion found that background infusion was significantly associated with increased fentanyl consumption and a higher incidence of opioid-related side effects [40]. Given these findings, future studies should consider using PCA without background infusion to more accurately assess the analgesic and safety effects of adding paracetamol/NSAIDs to opioid-based analgesia. Third, this study excluded patients with hepatic or renal impairment. While co-administration of acetaminophen, ibuprofen, and fentanyl has been shown to be safe in several clinical settings [6], the pharmacokinetic (PK) and pharmacodynamic (PD) interactions between these drugs should be carefully considered, especially in the elderly or patients with hypoalbuminemia, due to the potential for altered drug metabolism and adverse drug interactions. Lastly, although paracetamol and NSAIDs are theoretically hypothesized to exert antitumor effects via COX-2 inhibition, oncologic outcomes such as cancer recurrence and mortality were not assessed in this study. These outcomes are beyond the scope of the current work and should be interpreted with caution. Future prospective trials are warranted to evaluate their long-term oncologic impact when used as adjuncts to opioids for postoperative analgesia.

## 5. Conclusions

In conclusion, the addition of intravenous acetaminophen and ibuprofen to opioid-based analgesia significantly reduced immediate postoperative pain, opioid consumption, and the need for additional analgesics after oncoplastic breast surgery, without increasing adverse effects. This multimodal analgesic strategy is effective and safe. It may improve postoperative pain control, reduce opioid reliance, and potentially mitigate perioperative immune suppression.

## Figures and Tables

**Table 1:** Demographics, clinical, and laboratory data.

Variables	Intervention Group (n = 39)	Control Group (n = 40)	*p* Value
Age (years)	50.1 ± 10.4	48.4 ± 12.3	0.515
Body mass index (kg/m^2^)	23.2 ± 3.4	23.5 ± 3.2	0.686
Hypertension	10 (25.6%)	6 (15.0%)	0.239
Diabetes mellitus	1 (2.6%)	3 (7.5%)	0.317
ASA class I/II/III	25/13/1	26/11/3	0.556
Preoperative albumin (g/dL)	4.5 (4.3, 4.6)	4.5 (4.3, 4.7)	0.670
Preoperative creatinine (mg/dL)	0.68 (0.62, 0.79)	0.68 (0.62, 0.75)	0.424
Preoperative aspartate transaminase (IU/L)	24 (20, 29)	24 (20, 29)	0.918
Preoperative alanine transferase (IU/L)	20 (14, 25)	18 (15, 32)	0.988
Operation type			
Tissue expander procedures	4 (10.3%)	4 (10.0%)	0.485
Implant procedures	12 (30.8%)	9 (22.5%)	
Tissue expander removal & implant insertion	13 (33.3%)	14 (35.0%)	
Implant exchange & capsulectomy	10 (25.6%)	10 (25.0%)	
Implant procedure & simple mammoplasty	0	3 (7.5%)	
Duration of operation (min)	105.8 ± 46.3	110.4 ± 54.4	0.697
Duration of anesthesia (min)	135.7 ± 49.5	141.1 ± 56.3	0.659
Remifentanil (mcg/kg/min)	0.06 (0.05, 0.07)	0.05 (0.05, 0.07)	0.211

Values are mean ± standard deviation, number (percent), or median (interquartile range). ASA class, American Society of Anesthesiologists physical status classification.

**Table 2:** Postoperative pain intensity.

Variables	Intervention Group (n = 39)	Control Group (n = 40)	*p* Value
At 1 h after surgery			
NRS at rest	2 (1, 2)	2 (1, 2)	0.789
NRS during movement	2 (2, 2)	2 (2, 3)	0.040
At 6 h after surgery			
NRS at rest	3 (2, 4)	3 (2, 4)	0.319
NRS during movement	6 (4, 7)	5 (4, 7)	0.758
At 24 h after surgery			
NRS at rest	2 (0, 3)	2 (1, 2)	0.630
NRS during movement	4 (3, 5)	3 (2, 5)	0.353
At 48 h after surgery			
NRS at rest	1 (0, 2)	1 (0, 1)	0.398
NRS during movement	2 (2, 3)	2 (1, 2)	0.314

Values are median (interquartile range). NRS, numerical rating scale (0 = no pain, 10 = worst pain).

**Table 3:** Cumulative fentanyl volume and additional analgesics requirement.

Variables	Intervention Group (n = 39)	Control Group (n = 40)	*p* Value
Cumulative fentanyl consumption via PCA (mcg)			
During 24 h after surgery	252.4 (186.7, 289.9)	299.7 (208.3, 366.6)	<0.001
During 48 h after surgery	482.4 (283.2, 548.0)	537.0 (390.9, 586.0)	0.001
Additional analgesic use beyond PCA, n (%)			
0–6 h after surgery			
Any rescue analgesic	22 (56.4%)	32 (80.0%)	0.024
Tramadol use	5 (12.8%)	9 (22.5%)	0.260
Acetaminophen	21 (53.8%)	28 (70.0%)	0.139
6–24 h after surgery			
Any rescue analgesic	16 (41.0%)	21 (52.5%)	0.307
Tramadol use	6 (15.4%)	5 (12.5%)	0.711
Acetaminophen use	14 (35.9%)	19 (47.5%)	0.296
24–48 h after surgery			
Any rescue analgesic	11 (28.2%)	12 (30.0%)	0.861
Tramadol use	1 (2.6%)	1 (2.5%)	0.986
Acetaminophen use	10 (25.6%)	12 (30.0%)	0.666

Values are median (interquartile range) or number (%).

**Table 4:** Analgesic-related side effects and hospital stay.

Variables	Intervention Group (n = 39)	Control Group (n = 40)	*p* Value
Nausea	17 (43.6%)	14 (35.0%)	0.434
Vomiting	5 (12.8%)	3 (7.5%)	0.433
Headache	2 (5.1%)	3 (7.5%)	0.665
Hypotension	5 (12.8%)	4 (10.0%)	0.693
Dizziness	4 (10.3%)	10 (25.0%)	0.086
Pruritus	0	1 (2.5%)	0.320
Postoperative hospital stay (days)	3 (2, 3)	3 (2, 4)	0.275

Values are the number (%) or median (interquartile range).

## Data Availability

The datasets generated during and/or analyzed during the current study are available from the corresponding author on reasonable request.

## Abbreviations

PCA	Patient-Controlled Analgesia
PROSPECT	Procedure-Specific Postoperative Pain Management
NSAIDs	Nonsteroidal Anti-Inflammatory Drugs
ERAS	Enhanced Recovery After Surgery
ASA	American Society of Anesthesiologists
BIS	Bispectral Index
NRS	Numerical Rating Scale
COX	Cyclooxygenase

## References

[1] Bray F., Laversanne M., Sung H., Ferlay J., Siegel R.L., Soerjomataram I., Jemal A. (2024). Global cancer statistics 2022: GLOBOCAN estimates of incidence and mortality worldwide for 36 cancers in 185 countries. CA Cancer J. Clin..

[2] Cata J.P., Corrales G., Speer B., Owusu-Agyemang P. (2019). Postoperative acute pain challenges in patients with cancer. Best Pract. Res. Clin. Anaesthesiol..

[3] Jacobs A., Lemoine A., Joshi G.P., Van de Velde M., Bonnet F., the PROSPECT Working Group collaborators (2020). PROSPECT guideline for oncological breast surgery: A systematic review and procedure—Specific postoperative pain management recommendations. Anaesthesia.

[4] Temple-Oberle C.M., Shea-Budgell M.A.M., Tan M., Semple J.L.M., Schrag C., Barreto M., Blondeel P.M., Hamming J., Dayan J., Ljungqvist O.M. (2017). Consensus Review of Optimal Perioperative Care in Breast Reconstruction: Enhanced Recovery after Surgery (ERAS) Society Recommendations. Plast. Reconstr. Surg..

[5] Ong C.K.S., Seymour R.A., Lirk P., Merry A.F. (2010). Combining Paracetamol (Acetaminophen) with Nonsteroidal Antiinflammatory Drugs. Anesth. Analg..

[6] Lee H.-J., Choi S., Yoon S., Yoon S., Bahk J.-H. (2024). Effect of an intravenous acetaminophen/ibuprofen fixed-dose combination on postoperative opioid consumption and pain after video-assisted thoracic surgery: A double-blind randomized controlled trial. Surg. Endosc..

[7] Retsky M., Rogers R., Demicheli R., Hrushesky W.J., Gukas I., Vaidya J.S., Baum M., Forget P., DeKock M., Pachmann K. (2012). NSAID analgesic ketorolac used perioperatively may suppress early breast cancer relapse: Particular relevance to triple negative subgroup. Breast Cancer Res. Treat..

[8] Shaji S., Smith C., Forget P. (2021). Perioperative NSAIDs and Long-Term Outcomes After cancer Surgery: A Systematic Review and Meta-analysis. Curr. Oncol. Rep..

[9] Horine S.V., Rakesh N., Nadav D., Gulati A. (2025). Perioperative Pain Management in Patients with Cancer-Related Pain: A Narrative Review. Anesth. Analg..

[10] Paul A.K., Smith C.M., Rahmatullah M., Nissapatorn V., Wilairatana P., Spetea M., Gueven N., Dietis N. (2021). Opioid Analgesia and Opioid-Induced Adverse Effects: A Review. Pharmaceuticals.

[11] Neuman M.D., Bateman B.T., Wunsch H. (2019). Inappropriate opioid prescription after surgery. Lancet.

[12] Hoffmann J., Wallwiener D. (2009). Classifying breast cancer surgery: A novel, complexity-based system for oncological, oncoplastic and reconstructive procedures, and proof of principle by analysis of 1225 operations in 1166 patients. BMC Cancer.

[13] Salaffi F., Stancati A., Silvestri C.A., Ciapetti A., Grassi W. (2004). Minimal clinically important changes in chronic musculoskeletal pain intensity measured on a numerical rating scale. Eur. J. Pain.

[14] Clephas P.R., Orbach-Zinger S., Gosteli-Peter M.A., Hoshen M., Halpern S., Hilber N.D., Leo C., Heesen M. (2025). Regional analgesia techniques for postoperative pain after breast cancer surgery: A network meta-analysis. Cochrane Database Syst. Rev..

[15] Tohme S., Simmons R.L., Tsung A. (2017). Surgery for cancer: A trigger for metastases. Cancer Res..

[16] Cole S.W., Nagaraja A.S., Lutgendorf S.K., Green P.A., Sood A.K. (2015). Sympathetic nervous system regulation of the tumour microenvironment. Nat. Rev. Cancer.

[17] Angele M.K., Faist E. (2002). Clinical review: Immunodepression in the surgical patient and increased susceptibility to infection. Crit. Care.

[18] O’nEill A., Lirk P. (2022). Multimodal Analgesia. Anesthesiol. Clin..

[19] Martinez V., Beloeil H., Marret E., Fletcher D., Ravaud P., Trinquart L. (2016). Non-opioid analgesics in adults after major surgery: Systematic review with network meta-analysis of randomized trials. Br. J. Anaesth..

[20] Cho J.S., Lee M.-H., Kim S.I., Park S., Park H.S., Oh E., Lee J.H., Koo B.-N. (2017). The Effects of Perioperative Anesthesia and Analgesia on Immune Function in Patients Undergoing Breast Cancer Resection: A Prospective Randomized Study. Int. J. Med. Sci..

[21] Boland J.W., Pockley A.G. (2017). Influence of opioids on immune function in patients with cancer pain: From bench to bedside. Br. J. Pharmacol..

[22] Tripolt S., Neubauer H.A., Knab V.M., Elmer D.P., Aberger F., Moriggl R., Fux D.A. (2021). Opioids drive breast cancer metastasis through the δ-opioid receptor and oncogenic STAT3. Neoplasia.

[23] Klifto K.M., Elhelali A., Payne R.M., Cooney C.M., Manahan M.A., Rosson G.D. (2021). Perioperative systemic nonsteroidal anti-inflammatory drugs (NSAIDs) in women undergoing breast surgery. Cochrane Database Syst. Rev..

[24] Boland G.P., Butt I.S., Prasad R., Knox W.F., Bundred N.J. (2004). COX-2 expression is associated with an aggressive phenotype in ductal carcinoma in situ. Br. J. Cancer.

[25] Reader J., Holt D., Fulton A. (2011). Prostaglandin E2 EP receptors as therapeutic targets in breast cancer. Cancer Metastasis Rev..

[26] Moris D., Kontos M., Spartalis E., Fentiman I.S. (2016). The Role of NSAIDs in Breast Cancer Prevention and Relapse: Current Evidence and Future Perspectives. Breast Care.

[27] Lu Y.-C., Chen P.-T., Lin M.-C., Lin C.-C., Wang S.-H., Pan Y.-J. (2021). Nonsteroidal Anti-Inflammatory Drugs Reduce Second Cancer Risk in Patients With Breast Cancer: A Nationwide Population-Based Propensity Score-Matched Cohort Study in Taiwan. Front. Oncol..

[28] Forget P., Vandenhende J., Berliere M., Machiels J.-P., Nussbaum B., Legrand C., De Kock M. (2010). Do intraoperative analgesics influence breast cancer recurrence after mastectomy? A retrospective analysis. Anesth. Analg..

[29] Retsky M., Demicheli R., Hrushesky W.J., Forget P., De Kock M., Gukas I., Rogers R.A., Baum M., Sukhatme V., Vaidya J.S. (2013). Reduction of Breast Cancer Relapses with Perioperative Non-Steroidal Anti-Inflammatory Drugs: New Findings and a Review. Curr. Med. Chem..

[30] Ayoub S.S. (2021). Paracetamol (acetaminophen): A familiar drug with an unexplained mechanism of action. Temperature.

[31] Camu F., Borgeat A., Heylen R.J., Viel E.J., Boye M.E., Cheung R.Y. (2016). Parecoxib, propacetamol, and their combination for analgesia after total hip arthroplasty: A randomized non-inferiority trial. Acta Anaesthesiol. Scand..

[32] Daniels S.E., Playne R., Stanescu I., Zhang J., Gottlieb I.J., Atkinson H.C. (2019). Efficacy and Safety of an Intravenous Acetaminophen/Ibuprofen Fixed-dose Combination After Bunionectomy: A Randomized, Double-blind, Factorial, Placebo-controlled Trial. Clin. Ther..

[33] Gottlieb I.J., Gilchrist N., Carson S., Stanescu I., Atkinson H. (2021). Extending the safety profile of the post-operative administration of an intravenous acetaminophen/ibuprofen fixed dose combination: An open-label, multi-center, single arm, multiple dose study. Biomed. Pharmacother..

[34] Tena-Garitaonaindia M., Rubio J.M., Martínez-Plata E., Martínez-Augustin O., de Medina F.S. (2025). Pharmacological bases of combining nonsteroidal antiinflammatory drugs and paracetamol. Biomed. Pharmacother..

[35] Ohnesorge H., Bein B., Hanss R., Francksen H., Mayer L., Scholz J., Tonner P.H. (2009). Paracetamol versus metamizol in the treatment of postoperative pain after breast surgery: A randomized, controlled trial. Eur. J. Anaesthesiol..

[36] De Oliveira G.S., Rodes M.E., Bialek J., Kendall M.C., McCarthy R.J. (2017). Single dose systemic acetaminophen to improve patient reported quality of recovery after ambulatory segmental mastectomy: A prospective, randomized, double-blinded, placebo controlled, clinical trial. Breast J..

[37] Mitchell A., McCrea P., Inglis K., Porter G. (2012). A Randomized, Controlled Trial Comparing Acetaminophen Plus Ibuprofen versus Acetaminophen Plus Codeine Plus Caffeine (Tylenol 3) after Outpatient Breast Surgery. Ann. Surg. Oncol..

[38] van Helmond N., Steegers M.A., Moor G.P.F.-D., Vissers K.C., Wilder-Smith O.H. (2016). Hyperalgesia and Persistent Pain after Breast Cancer Surgery: A Prospective Randomized Controlled Trial with Perioperative COX-2 Inhibition. PLoS ONE.

[39] Graham L.A., Illarmo S.S., Wren S.M., Odden M.C., Mudumbai S.C. (2024). Variations in Current Practice and Protocols of Intraoperative Multimodal Analgesia: A Cross-Sectional Study Within a Six-Hospital US Health Care System. Anesth. Analg..

[40] Jung H., Lee K.H., Jeong Y., Lee K.H., Yoon S., Kim W.H., Lee H. (2020). Effect of Fentanyl-Based Intravenous Patient-Controlled Analgesia with and without Basal Infusion on Postoperative Opioid Consumption and Opioid-Related Side Effects: A Retrospective Cohort Study. J. Pain Res..

